# Excess costs of mental disorders by level of severity

**DOI:** 10.1007/s00127-022-02298-8

**Published:** 2022-05-31

**Authors:** Hannah König, Hans-Helmut König, Jürgen Gallinat, Martin Lambert, Anne Karow, Judith Peth, Holger Schulz, Alexander Konnopka

**Affiliations:** 1 Department of Health Economics and Health Services Research, Center for Psychosocial Medicine, University Medical Center Hamburg-Eppendorf, Martinistraße 52, 20246 Hamburg, Germany; 2 Department of Psychiatry and Psychotherapy, Center for Psychosocial Medicine, University Medical Center Hamburg-Eppendorf, Hamburg, Germany; 3 Department of Medical Psychology, Center for Psychosocial Medicine, University Medical Center Hamburg-Eppendorf, Hamburg, Germany

**Keywords:** Mental disorders, Cost of Illness, Disease severity, Health Services Research

## Abstract

**Introduction:**

Mental disorders are highly prevalent in Germany, and associated with decreased quality of life for those affected as well as high economic burden for the society. The purpose of this study was to determine the excess costs of mental disorders and to examine how these differ with respect to disease severity.

**Methods:**

We analyzed mean 6-month costs using the baseline data from the RECOVER trial in Hamburg, Germany, which evaluates an innovative stepped-care model for mental disorders. Four severity levels were classified based on the current level of mental illness, loss of functioning, and psychiatric diagnosis. In this work, direct costs (outpatient, inpatient, and social/informal care) and indirect costs (sick leave, unemployment, and early retirement) were estimated using interview-based data on health care utilization and productivity losses. Excess costs were determined by matching a comparison group of the German general population without mental disorders. Group differences in sociodemographic covariates and somatic comorbidities were balanced using entropy balancing. Excess costs by severity levels were estimated using generalized linear models (GLM) with gamma distribution and log-link function.

**Results:**

Overall, the RECOVER group included *n* = 816 and the comparison group included *n* = 3226 individuals. Mean total 6-month excess costs amounted to 19,075€, with higher indirect excess costs (13,331€) than direct excess costs (5744€) in total excess costs. The excess costs increased with increasing disease severity, ranging from 6,123€ with mild disease severity (level 1) to 31,883€ with severe mental illness (level 4). Indirect excess costs ranged from 5612€ in level 1 to 21,399€ in level 4, and were statistically significant for all disease severity levels. In contrast, direct excess costs were only statistically significant for the levels 2 to 4, and ranged from 511€ in level 1 to 10,485€ in level 4. The main cost drivers were hospital stays (level 2–4), sick leave and unemployment (all levels), and early retirement (level 3–4).

**Discussion:**

Mental disorders are associated with high costs that increase with the level of disease severity, which was also shown for individual ICD-10 diagnosis groups. Due to their influence on costs, indirect costs and disease severity levels should be considered in future cost-of-illness studies of mental disorders.

**Clinical trial registration:**

clinicaltrials.gov, trial registration number NCT03459664.

**Supplementary Information:**

The online version contains supplementary material available at 10.1007/s00127-022-02298-8.

## Introduction

Mental disorders are associated with a high disease burden, including reduced quality of life and social functioning, as well as increased costs for society [1, 2]. In Germany, the total costs of mental disorders are estimated to be about 147 billion Euros in 2015, including direct costs due to health care service utilization, spending for social security, and indirect costs due to lost or reduced productivity [3]. With a 12-month-prevalence of 27.8%, about one third of the German adult population is affected by at least one mental disorder each year [4]. However, only 18.9% of the individuals with mental disorders (12-month diagnosis) sought for any health care treatment related to mental health within 1 year [5].

By international standards, the German health care system generally provides universal access, and a high quality of care [6]. Yet, there are shortcomings in care with respect to the treatment of mental disorders. In general, the outpatient and inpatient sectors are strictly separated, which limits the continuity of treatment for individuals with mental disorders [7]. Long waiting times, especially in the outpatient sector, encourage a shift to the cost-intensive inpatient sector [8]. In this context, the German mental health care system is particularly challenging, as a large number of different health service providers, funded by different authorities, is involved [9]. Furthermore, for mental disorders such as schizophrenia, which are associated with severe courses of illness, there are fewer psychotherapeutic services available than recommended in clinical practice guidelines [7]. New care approaches aim to overcome the existing structural deficits in the health care system to meet the demand for more patient-oriented, resource-efficient treatment options. The German Association for Psychiatry, Psychotherapy and Psychosomatics (Deutsche Gesellschaft für Psychiatrie und Psychotherapie, Psychosomatik und Nervenheilkunde e. V.—DGPPN) calls among others for treatment of mental disorders based on the level of disease severity, as provided by stepped care approaches [10]. The stepped care approach aims to improve care while being resource efficient by aligning and constantly adjusting the intensity of care with the disease severity, starting with the least intensive approach [11].

Cost-of-illness studies of mental disorders provide information about the economic impact on society, which can be valuable for health policy decisions. So far, few studies have analyzed the costs of mental disorders as a group in Germany, most recently, the study by Gustavsson and colleagues [12]. This study provides comprehensive cost estimates of mental and neurological disorders for several European countries, which were also used for more recent reports on the economic burden of mental disorders [3, 12]. However, the data for the included diagnosis groups stem from different data sources that originated in 2010 or earlier [12]. Excess costs are generally reported less frequently, and to our knowledge, excess costs of mental disorders have not been estimated for the German health care system yet. Within the EU, the latest findings on excess costs of mental disorders were reported from Austria [13]. Excess costs are calculated as the difference in mean costs of a group with a particular disease and a control group without that disease. The advantage of this approach is that it also captures costs that cannot be directly related to a disease, and thus cost-of-illness can be measured more precisely [14, 15]. More recent studies from Germany have estimated the cost-of-illness of specific diagnosis groups, including social anxiety disorder, depression, alcohol-dependence and schizophrenia [16–19]. Since there is in most cases no differentiation between diagnosis groups in the provision of health care services for mental disorders, cost estimates for mental disorders as a whole have the advantage that they are much closer to the actual healthcare system. In addition, comorbid mental disorders can be addressed comprehensively. This study contributes to the existing body of research by providing actual estimates of the direct and indirect excess costs of mental disorders in Germany. Further, excess costs by different levels of disease-severity are estimated to provide information on the extent to which severity levels differ in terms of health care utilization and productivity losses, and thus economic burden.

## Methods

### Study design

Data on individuals with mental disorders were obtained from the randomized-controlled trial (RCT) RECOVER, conducted at the University Medical Center Hamburg-Eppendorf in Northern Germany. In the RECOVER care model, several innovative and evidence-based care components such as case management, assertive community treatment, and e-therapy were combined in a cross-sectoral, stepped care approach and compared to regular care with respect to patient-relevant outcomes, costs, and effectiveness [20]. For our analysis, we used the baseline data (*n* = 889), which was collected from 2018 to 2019 by clinically trained interviewers. We matched a control group using a dataset representative of the German general population in age, sex, residence state, and education level (*n* = 5005) [21]. The data of the control group were collected via telephone interviews and contains general information on the sample like sociodemographic variables or comorbidities, as well as information on the resource utilization. More details on the data sources are published elsewhere [20, 21].

### Study sample

Many study participants with mental disorders were directly recruited at the University Medical Center Hamburg-Eppendorf, while other recruitment channels were external partners such as primary care physicians or psychotherapists, or the website of the RECOVER study. Since home treatment was a part of the RECOVER intervention, the study participants were recruited from the catchment area of the study center (≤ 8 km distance). Furthermore, study participants were eligible for inclusion if they were ≥ 16 years old and insured in one of the participating statutory health insurance funds, which together account for a population share of approximately 38%.

We excluded participants with missing values with respect to sociodemographic and clinical covariates (*n* = 38), health care utilization and productivity losses (*n* = 35) from the analysis, resulting in a final sample of *n* = 816 individuals with mental disorders. Among these were *n* = 56 individuals with mild disease severity (level 1), *n* = 306 individuals with moderate disease severity (level 2), *n* = 294 individuals with moderate to severe disease severity (level 3), and *n* = 160 individuals with severe mental illness (level 4).

As for the control group, we further restricted the data to individuals without self-reported medical diagnosis of any mental disorder (exclusion of *n* = 985 participants), with statutory health insurance (exclusion of *n* = 685 participants), and without missing values (exclusion of *n* = 109 participants), resulting in a final sample of *n* = 3226 individuals without mental disorders.

### Assessment of the psychiatric diagnosis

Clinically trained interviewers conducted a thorough assessment of the psychiatric diagnosis using the structured clinical interview for DSM-IV (SCID-I/II) [22] and diagnosis-specific questionnaires (for details see Table S1) [23–42]. Participants with any primary diagnosis of a mental disorder according to the International Statistical Classification of Diseases and Related Health Problems—10th Revision (ICD-10) were included, except for study participants with a primary diagnosis in the spectrum of organic, including symptomatic, mental disorders (ICD-10 F0) or mental and behavioral disorders due to psychoactive substance use (ICD-10 F1). The following primary diagnoses were included: Schizophrenia, schizotypal and delusional disorders (ICD-10 F2), mood disorders (ICD-10 F3), neurotic, stress-related and somatoform disorders (ICD-10 F4), behavioral syndromes associated with physiological disturbances and physical factors (ICD-10 F5), disorders of adult personality and behavior (ICD-10 F6), disorders of psychological development (ICD-10 F8) or behavioral and emotional disorders with onset usually occurring in childhood and adolescence (ICD-10 F9).

### Assessment of the severity level

In this study, four severity levels were distinguished as classified for the stepped-care treatment in the RECOVER study, which was based on the psychiatric diagnosis, the current level of mental illness (Clinical Global Impressions-Severity scale, CGI-S [43]), and the loss of functioning (Global Assessment of Functioning, GAF [44]), for further details see Table 1 and the study protocol of the RECOVER study [20]. Only patients with severe mental illness and a diagnosis of psychosis or borderline personality disorder were assigned to severity level 4, as they were included in integrated care models for these disorders. If there were discrepancies between the three measures, the GAF and CGI-S were leading, and in the rare case that these measures differed, the GAF. For severity level 4, it was sufficient if either the GAF or the CGI-S fulfilled the criteria for a severe mental illness.

**Table 1:** Severity levels in the RECOVER RCT

Severity level	CGI-S *Current level of mental illness*	GAF *Loss of functioning*	Primarily psychiatric diagnoses (ICD-10)
1 *mild disease severity*	1–3 in the last 4 weeks	61–100: no or mild symptoms in the last 4 weeks	Mood disorders (ICD-10 F32), neurotic, stress-related and somatoform disorders (ICD-10 F40, F41, F43.2, F45), behavioral and emotional disorders with onset usually occurring in childhood and adolescence (ICD-10 F90)
2 *moderate disease severity*	3–4 in the last 4 weeks	51–60: moderate symptoms in the last 4 weeks	Mood disorders (ICD-10 F32), neurotic, stress-related and somatoform disorders (ICD-10 F40, F41, F42, F43.1, F43.2, F45), Behavioral syndromes associated with physiological disturbances and physical factors (ICD-10 F50), Behavioral and emotional disorders with onset usually occurring in childhood and adolescence ICD-10 F90)
3 *moderate to severe disease severity*	4–6 in the last 4 weeks	31–50: serious symptoms or impairments in the last 4 weeks	Schizophrenia, schizotypal and delusional disorders (ICD-10 F20, F22, F23, F25), mood disorders (ICD-10 F31, F33), neurotic, stress-related and somatoform disorders (ICD-10 F41, F42, F43.1, F45), behavioral syndromes associated with physiological disturbances and physical factors (ICD-10 F50), Disorders of adult personality and behavior (ICD-10 F60, F61)
4 *severe mental illness*	5–7 in the last 4 weeks	≤ 50: serious or major impairments over at least six months	Schizophrenia, schizotypal and delusional disorders (ICD-10 F20, F22, F23, F25), mood disorders (ICD-10 F31, F32.3, F33.3), disorders of adult personality and behavior (ICD-10 F60)

*CGI-S* Clinical Global Impressions-Severity scale, *GAF* Global Assessment of Functioning, *ICD-10* International Statistical Classification of Diseases and Related Health Problems—10th Revision

### Cost assessment

We analyzed 6-month direct and indirect costs on the basis of self-reported resource utilization, assessed for the 6 months before the interview using modified versions of the FIMA questionnaire (‘Fragebogen zur Erhebung von Gesundheitsleistungen im Alter’—Questionnaire for Health-Related Resource Use in an Elderly Population) and FIMPsy questionnaire (‘Fragebogen zur Inanspruchnahme medizinischer und nicht medizinischer Versorgungsleistungen bei psychischen Erkrankungen’—Questionnaire for the Assessment of Medical and non Medical Resource Utilization in Mental Disorders) [45, 46]. In general, the costs were estimated by multiplying self-reported resource use with published unit costs.

Direct costs included outpatient, inpatient, and social/informal care services, which were valued with published unit costs for Germany [47, 48]. Outpatient services encompassed physicians, outpatient clinic/psychiatric outpatient department, and other health care professionals. In the RECOVER baseline data, outpatient treatment in the hospital was surveyed separately for general and psychiatric services, contrary to the dataset of the control group. Therefore, the data were combined in the cost category ‘outpatient clinic/psychiatric outpatient department’. For outpatient clinic/psychiatric outpatient department and radiologists, we used the arithmetic mean of the other physicians’ unit costs since no published unit costs were available. Inpatient services encompassed hospital/day-care and rehabilitation. Hospital/day care encompassed day care and inpatient stays in general or psychiatric hospitals. For full inpatient stays (> 1 day in hospital/rehabilitation), we subtracted 1 day from the number of days reported, as the number of overnight stays are reimbursed in Germany. Social/informal care services encompassed professional home help and help from family or friends (informal care). Informal care was assessed by asking the study participants about the number of days they received help from family and friends in the last 6 months, and the average duration of this contact per day. The information on total duration (in hours) was then valued monetarily with average published unit costs, representing substitution costs of a comparable professional occupation [47]. The following services were assessed for the group of individuals with mental disorders, but were not available for the control group, and consequently not included in the cost analysis: Medication, sheltered/supported accommodation, and services for participation in working life.

Indirect costs included productivity losses due to sick leave, unemployment, and early retirement and were estimated with the human capital approach. The human capital approach assesses productivity losses to society by taking the entire period of lost working force due to morbidity or death into account, and evaluates indirect costs with income losses [49]. Another commonly used approach would be the friction cost approach, which only assesses the costs incurred in the time period until the replacement by a new employee, including training and recruitment-related expenses [50]. In both approaches, productivity losses due to unemployment are generally not directly evaluated. Mental disorders are associated with higher unemployment rates than in the general population [51, 52]. In this analysis, we wanted to assess the differences in unemployment rates between individuals with mental disorders and individuals without mental disorders. For this purpose, we have also valued unemployment in monetary terms.

The basic approach was to multiply the productivity losses by average daily wages for full-time and part-time employees. In particular, the average monthly gross income for full-time and part-time work was supplemented by the employer's social insurance contributions, and calculated on a daily basis after deduction of vacation days and public holidays, resulting in 265.53 € per day [53, 54]. We estimated the costs of sick leave for individuals who reported to be working either in full-time or part-time by valuing monetarily the number of self-reported days spent on sick leave in the last 6 months. The costs of early retirement were estimated if persons indicated that they received a disability pension at the time of the interview. In Germany, individuals receiving full disability pension are allowed to work a maximum of two hours per day, which corresponds to 6 h or 75% of lost productivity in a regular 8-h working day [55]. The amount of productivity losses due to the disability pension was not assessed. Therefore, we assumed productivity losses of 75% for full disability pension, which equals 97.5 days of lost productivity for 6 months. In this study, the unemployment rates for individuals with mental disorders were much higher compared to individuals without mental disorders. Therefore, we also valued the costs of unemployment monetarily by valuing assumed productivity losses of 100% (130 days) monetarily if individuals indicated that they were unemployed at the time of the interview. All unit costs were inflated to the year 2019 using the consumer price index. Table S2 provides further details on the cost categories included and their respective unit costs.

### Statistical analysis

Before the analysis of excess costs, differences in sociodemographic covariates and somatic comorbidities between the group of individuals with mental disorders and the control group were balanced to account for potential confounding. Balancing of the groups was performed with entropy balancing (EB) using the corresponding software package in Stata [56]. EB is a method that assigns a weight to each observation in the control group to achieve covariate balance between groups, in this study with respect to the mean and variance of all covariates included in the EB model [57]. EB allows to balance many relevant socioeconomic and clinical covariates at the same time, and can thus be used to estimate the average treatment effect on the treated [57]. The following covariates were reweighted in the control group to balance the control group and the group of individuals with mental disorders: age, gender, marital status (three categories), school level (five categories), education level (three categories), body mass index (five categories following the World Health Organization [58]), and somatic comorbidities according to the ICD-10 chapters II, IV, and IX–XIII. After EB, the included covariates are expected to be almost equally distributed in mean and variance between the group of individuals with mental disorders and the control group. See Table 2 for the sample characteristics of the groups and more details on the included covariates, and see Table S3 in the supplement for the sample characteristics before balancing.

**Table 2:** Sample characteristics of the matched groups

Covariates	Individuals with mental disorders (*n* = 816)	Matched control group without mental disorders (*n* = 816^1^)
Age (in years, mean)	36.9	36.9
Gender (female, %)	59.9	59.9
Marital status (%)		
Single	53.8	53.8
Married/having a partner	38.6	38.6
Divorced/living separated	7.6	7.6
School level (%)		
No school-leaving diploma	3.4	3.4
Special-needs school (Förderschule)	0.5	0.5
Secondary general school (Hauptschulabschluss)	14.5	14.5
Secondary school (Mittlerer Schulabschluss)	28.9	28.9
Academic secondary school ((Fach-)Abitur)	52.7	52.7
Education level (%)		
No graduation	37.0	37.0
Completed vocational training	40.7	40.7
University diploma	22.3	22.3
Body mass index (%)		
Underweight (< 18.5)	6.3	6.2
Normal weight (18.5–24.9)	52.8	52.8
Overweight (25.0–29.9)	23.9	23.9
Class I obesity (30–34.9)	11.2	11.2
Class II–III obesity (≥ 35)	5.9	5.9
Neoplasms^2^ (%)	1.6	1.6
Endocrine, nutritional and metabolic diseases^3^ (%)	15.2	15.2
Diseases of the circulatory system^4^ (%)	11.8	11.8
Diseases of the respiratory system^5^ (%)	16.3	16.3
Diseases of the digestive system^6^ (%)	6.9	6.9
Diseases of the skin and subcutaneous tissue^7^ (%)	5.9	5.9
Diseases of the musculoskeletal system and connective tissue^8^ (%)	13.2	13.3

For all covariates, there are not statistically significant differences between the group of individuals with mental disorders and the matched control group without mental disorders

^1^*n* = 3226 individuals in the control group were down weighted to match the individuals with mental disorders

^2^ICD-10 chapter II

^3^ICD-10 chapter IV

^4^ICD-10 chapter IX

^5^ICD-10 chapter X

^6^ICD-10 chapter XI

^7^ICD-10 chapter XII

^8^ICD-10 chapter XIII

We estimated costs using generalized linear models (GLM) with a gamma distribution and log-link function to account for the distribution of cost data. Each cost category was analyzed in a GLM, with the respective cost category as dependent variable and the group assignment (individuals with/without mental disorders) as independent variable. The groups were adjusted for observable factors by including the EB weights in the GLM. For each group, the weighted mean costs were calculated. The excess costs were estimated as average marginal effects of the groups, and considered to be statistically significant at a *p* value < 0.05. The excess costs were further estimated for each severity level using the previously calculated EB weights to balance the respective severity level group and the control group. In addition, subgroup analyses were performed for the most frequent primary diagnosis groups according to the ICD-10, as these were represented differently in the severity levels. Since the subdivision according to main diagnosis led to small subgroups in some cases, we only analyzed subgroups with at least 25 observations. For severity level 1, no subgroup analyses were performed. In severity levels 2 and 3, the diagnosis groups neurotic, stress-related and somatoform disorders (ICD-10 F4), mood disorders (ICD-10 F3), and disorders of adult personality and behavior (ICD-10 F6) were analyzed, and in level 4, the diagnosis groups disorders of adult personality and behavior (ICD-10 F6) and schizophrenia, schizotypal and delusional disorders (ICD-10 F2). For better comparability across subgroups, the control groups were adjusted using the same EB weights as in the primary analysis. All analyses were performed in Stata version 16.

### Sensitivity analyses

In a first sensitivity analysis, we conducted an unweighted analysis of excess costs to test the influence of the group balancing on costs. In this study, unemployment was considered when calculating indirect costs, which is quite uncommon in cost-of-illness studies. Therefore, we excluded the indirect costs of unemployment in a second sensitivity analysis to test their influence on the total and indirect excess costs.

## Results

### Sample characteristics

Among the individuals with mental disorders, 46.8% were diagnosed with mood disorders (ICD-10 F3), in 90.3% of the cases major depression, followed by 22.4% diagnosed with neurotic, stress-related and somatoform disorders (ICD-10 F4), 21.6% diagnosed with disorders of adult personality and behavior (ICD-10 F6), 8.1% diagnosed with schizophrenia, schizotypal and delusional disorders (ICD-10 F2), 0.5% diagnosed with behavioral syndromes associated with physiological disturbances and physical factors (ICD-10 F5), 0.5% diagnosed with behavioral and emotional disorders with onset usually occurring in childhood and adolescence (ICD-10 F9), and 0.1% diagnosed with disorders of psychological development (ICD-10 F8). After the adjustment of the control group, the predefined covariates in the groups were almost equally distributed in mean and variance (see Table 2). In both groups, individuals were on average 36.9 years old, and the share of women (59.9%) was higher than that of men. The study participants were mostly single (53.8%) or married/having a partner (38.8%), and of normal weight (52.8%). Before the group adjustment, individuals in the control group were on average 54.4 years old, and the share of women (53.1%) was lower than after the adjustment; for more details, see Table S3.

However, the difference in unemployment rates was substantial: While unemployment was low at 2.9% in the control group, it was at 28.9% in the RECOVER sample. Within the group of individuals with mental disorders, the proportion of unemployment varied according to the main diagnosis, with about a quarter unemployed among individuals with mood disorders (ICD-10 F3) or neurotic, stress-related and somatoform disorders (ICD-10 F4), and about half of the individuals diagnosed with disorders of adult personality and behavior (ICD-10 F6) (42.6%) or schizophrenia, schizotypal and delusional disorders (ICD-10 F2) (50%). Table S4 provides details on the share of unemployment by severity levels as well as information on the quantity of health care services used.

### Excess costs

In total, the average 6-month excess costs of mental disorders amounted to 19,075 € (see Table 3). With 13,331 €, the share of indirect excess costs was considerably higher than the share of direct excess costs of 5744 €, mainly due to unemployment (8996 €). The main driver for direct excess costs was hospital/day care (5020 €). All estimated excess costs were statistically significant, with the exception of costs of services provided by other outpatient health care providers.

**Table 3:** Mean costs and excess costs of individuals with mental disorders (six months, in Euro 2019)

	Individuals with mental disorders (*n* = 816)		Matched control group (*n* = 816^1^)		Excess costs	95% confidence intervals	*p* value
Cost category	Mean costs	Standard error	Mean costs	Standard error			
Total costs	21,617	703	2542	314	19,075	17,566–20,583	< 0.001
Direct costs	6487	373	743	157	5744	4950–6537	< 0.001
Outpatient physicians	602	26	211	9	390	336–445	< 0.001
Outpatient other healthcare providers	51	6	49	5	2	− 13 to 18	0.768
Hospital/day care	5311	360	292	122	5020	4275–5764	< 0.001
Rehabilitation	92	28	28	13	64	3–125	0.040
Social/informal care	430	68	163	29	267	123–412	< 0.001
Indirect costs	15,130	522	1799	200	13,331	12,235–14,427	< 0.001
Sick leave	3179	237	466	45	2713	2241–3185	< 0.001
Unemployment	9983	548	988	180	8996	7865–10,126	< 0.001
Early retirement	1967	240	345	79	1622	1126–2118	< 0.001

^1^*n* = 3226 individuals in the control group were down weighted to match the individuals with mental disorders

The analysis of excess costs by severity levels showed that the excess costs of mental disorders increased with increasing disease severity (see Table 4; Table S5 shows the corresponding mean costs for each severity level). In total, the excess costs ranged between 6123 € in level 1 and 31,883 € in level 4. This trend could be seen for direct excess costs (511 € to 10,485 €) and indirect excess costs (5612 € to 21,399 €). Except for direct excess costs in level 1, all previously reported excess costs were statistically significant. Hospital/day care services were the main driver for direct excess costs in the severity levels 2–4, and the excess costs of outpatient physicians were also statistically significant higher for these severity levels. In addition, higher severity levels (3 and 4) incurred statistically significantly higher costs of social/informal care compared to individuals without mental disorders. Again, indirect excess costs were the main contributor to total excess costs for all severity levels. Although the costs of unemployment were statistically significantly increased in all severity levels, the excess costs in levels 1 and 2 were still considerably lower than in the case of a severe course of the disease. As for early retirement, none of the individuals with mild disease severity (level 1) received disability pension. Therefore, the average marginal effects of mental disorders compared to the control group were not estimable, and only descriptive early retirement excess costs were reported (− 345 €). For the higher severity levels, the excess costs of early retirement again were increasing with disease severity, but the estimates were statistically significant only in the levels 3 and 4. The excess costs of sick leave were statistically significant for all severity levels, peaked in level 2 (3426 €), and then decreased to 1858 € in level 4, which would be consistent with the findings that a smaller proportion of persons was working in severity levels 3 and 4.

**Table 4:** Excess costs by severity levels (6 months, in Euro 2019)

Cost category	Severity level 1 (*n* = 56)		Severity level 2 (*n* = 306)		Severity level 3 (*n* = 294)		Severity level 4 (*n* = 160)	
	Excess costs	95% CI	Excess costs	95% CI	Excess costs	95% CI	Excess costs	95% CI
Total costs	6123***	2940–9305	9841***	8008–11,674	24,182***	21,790–26,575	31,883***	28,860–34,907
Direct costs	511	− 490 to 1511	2417***	1620–3214	7624***	6216–9031	10,485***	8458–12,511
Outpatient physicians	85	− 9 to 179	302***	228–375	476***	384–569	509***	367–651
Outpatient other healthcare providers	− 33***	− 50 to − 15	− 1	− 19 to 18	5	− 16 to 27	15	− 25 to 54
Hospital	194	− 284 to 672	1966***	1242–2691	6701***	5343–8058	9459***	7458–11,460
Rehabilitation	65	− 119 to 249	78	− 40 to 197	52	− 18 to 122	58	− 58 to 175
Social/informal care	199	− 479 to 876	71	− 49 to 192	389**	152–626	444*	15–872
Indirect costs	5612***	2563–8661	7424***	5953–8895	16,559***	14,786–18,332	21,399***	19,229–23,568
Sick leave	2014***	1110–2917	3426***	2620–4232	2569***	1753–3385	1858***	915–2801
Unemployment	3944*	760–7127	3750***	2373–5127	11,693***	9758–13,628	15,840***	13,143–18,537
Early retirement	− 345^1^	–	247	− 214 to 708	2297***	1387–3206	3700***	2235–5165

95% CI—95% confidence intervals

*p < 0.05, **p < 0.01, ***p < 0.001

^1^Only descriptive estimates are reported, because none of the individuals with mental disorders received a disability pension

### Subgroup analysis

The analysis by ICD-10 diagnosis groups showed that the excess costs also increased with higher disease severity levels within diagnosis groups (see Table 5).

**Table 5:** Excess costs by severity levels and main diagnosis (6 months, in Euro 2019)

Cost category	Severity level 2		Severity level 3		Severity level 4	
	Excess costs	95% CI	Excess costs	95% CI	Excess costs	95% CI
Mood [affective] disorders (ICD-10 F3)						
Sample size	*n* = 203		*n* = 142		*n* = 14	
Total excess costs	10,622***	8354–12,889	24,195***	20,912–27,477	Not analyzed^1^	–
Direct excess costs	2734***	1746–3722	7617***	5657–9577	–	
Indirect excess costs	7888***	6070–9705	16,578***	14,080–19,075	–	
*Neurotic, stress-related and somatoform disorders (ICD-10 F4)*						
Sample size	*n* = 75		*n* = 77		*n* = 1	
Total excess costs	7513***	4367–10,660	25,067***	20,163–29,972	Not analyzed^1^	–
Direct excess costs	2155**	613–3698	9095***	5965–12,225	–	
Indirect excess costs	5358***	2856–7860	15,973***	12,541–19,404	–	
*Disorders of adult personality and behavior (ICD-10 F6)*						
Sample size	*n* = 25		*n* = 66		*n* = 84	
Total excess costs	11,840***	5443–18,236	25,073***	20,333–29,812	29,154***	24,878–33,429
Direct excess costs	920	− 395 to 2234	6703***	4163–9243	8907***	6250–11,565
Indirect excess costs	10,920***	5161–16,679	18,369***	14,740–21,999	20,246***	17,128–23,365
*Schizophrenia, schizotypal and delusional disorders (ICD-10 F2)*						
Sample size	*n* = 0		*n* = 5		*n* = 60	
Total excess costs	Not analyzed^1^	–	Not analyzed^1^	–	35,281***	31,074–39,489
Direct excess costs	–		–		11,100***	7886–14,314
Indirect excess costs	–		–		24,181***	21,170–27,192

^1^Only subgroups with a sufficient amount of individuals (*n* ≥ 25) were analyzed

*p < 0.05, **p < 0.01, ***p < 0.001

In severity level 2, the diagnosis group neurotic, stress-related and somatoform disorders (ICD-10 F4) incurred the lowest total excess costs (7513 €) compared to mood disorders (ICD-10 F3) (10,622 €) or disorders of adult personality and behavior (ICD-10 F6) (11,840 €). In contrast, the total excess costs of these diagnosis groups were similarly high in severity level 3 and ranged from 24,195 € to 25,073 €. In level 4, the total excess costs were higher for the diagnosis group schizophrenia, schizotypal and delusional disorders (ICD-10 F2) (35,281 €) than for disorders of adult personality and behavior (ICD-10 F6) (29,154 €). For all subgroups, indirect excess costs were statistically significant and with a share of at least 69% the main contributor to total excess costs. The only subgroup without statistically significant direct excess costs was the diagnosis group disorders of adult personality and behavior (ICD-10 F6) in severity level 2.

### Sensitivity analyses

The unadjusted excess costs in sensitivity analysis 1 (see Table S7) were in general slightly lower compared to the main analysis except for indirect excess costs, resulting from higher unemployment excess costs. In contrast to the main analysis, the unadjusted excess costs of rehabilitation and of social/informal care were not statistically significant, and the excess costs of other outpatient healthcare providers were negatively associated (− 17 €). In sensitivity analysis 2 without the costs of unemployment, the estimates were lower, but the direction and significance of the excess costs have not changed (see Table S7).

## Discussion

Overall, mental disorders showed high excess costs, which increased with the levels of disease severity. Direct costs were 8.7-times higher and indirect costs were 8.4-times higher than for individuals without mental disorders. The study sample was mainly composed of individuals diagnosed with mood disorders (ICD-10 F3), neurotic, stress-related and somatoform disorders (ICD-10 F4), disorders of adult personality and behavior (ICD-10 F6), and schizophrenia, schizotypal and delusional disorders (ICD-F2), although the recruitment of study participants was open to different mental disorders. However, these diagnosis groups also appear in public statistics among the most frequent diagnoses in specialist departments for psychiatry and psychotherapy in Germany [59]. Another explanation for the fact that mood disorders and neurotic, stress-related and somatoform disorders, with a total of almost 70%, represent the majority of the study sample would be that these disorders have the highest prevalence of all mental disorders in the German population with 12-month prevalence rates of 9.8% and 24.8%, respectively [4]. The analysis by disease severity showed that direct and indirect costs increased with increasing severity levels. With mild disease severity, the differences in direct costs were not significant. However, with mild disease severity, indirect costs due to unemployment and sick leave were already significantly higher compared to individuals without mental disorders, indicating that measures relating to the working life could already be useful at a mild severity level. The findings that the excess costs of early retirement were only significant in moderate to severe severity might reflect that these groups already have a chronic course of illness since it takes some time to receive an early retirement pension. Overall, moderate to severe mental disorders incurred the highest direct excess costs. Furthermore, inpatient stays were the main contributor to direct excess costs, and considerably high in severity levels 3 and 4. Therefore, acute treatment options and a shift to the outpatient sector could be a resource-efficient approach in the interest of the patient. The high excess costs for social/informal care services in the severity levels 3 and 4 show that these patient groups need support in everyday life, which is often provided by relatives. The resulting burden for relatives should be taken into account in future research, and might be reduced by better mental health care for severe mental disorders.

To our knowledge, no study has yet evaluated the excess costs of mental disorders in Germany. The excess costs of mental disorders in our study are quite high (direct and indirect costs of mental disorders were 8.7-times and 8.4-times higher, respectively) compared to estimates on the 12-month excess costs of mental disorders in Austria, which reported 1.4-times higher direct and 2.6-times higher indirect costs [13]. In our study, the high excess costs were also evident at the level of primary diagnosis groups: For the most represented disorders, mood disorders (ICD-10 F3) and neurotic, stress-related and somatoform disorders (ICD-10 F4), direct costs were both 7.6-times higher, respectively and indirect costs were 7.2-times and 6.5-times higher, respectively compared to the control group. These findings were considerably higher compared to previous findings from meta-analyses [60, 61]. An explanation for the high excess costs could be that many study participants were directly recruited from an inpatient stay at the University Medical Center Hamburg-Eppendorf, which is also reflected in the estimated excess costs for hospital/day care. There were also many referrals from primary care physicians, but they possibly only referred patients who were severely affected by the disease. Furthermore, although the majority of the study participants had a diagnosis of mood disorders or neurotic, stress-related and somatoform disorders, almost one third of the included study participants were diagnosed with disorders of adult personality and behavior (ICD-10 F6) or schizophrenia, schizotypal and delusional disorders (ICD-10 F2) disorders that are known to be associated with high costs [12]. The study by Frey reported 9.5-times higher annual total costs of patients with schizophrenia compared to individuals without schizophrenia in Germany [16]. These estimates were still lower than the 6-month total excess costs of schizophrenia, schizotypal and delusional disorders (ICD-10 F2) in this study, which were estimated to be 13.2-times higher compared to individuals without mental disorders. This could possibly be due to the fact that many participants in our analysis belonged to severity level 4, and thus often had a very severe disease manifestation or were close to inpatient treatment.

In this study, the share of indirect costs in total costs was equal to 70%, and thus higher than the share of direct costs. By contrast, population-based estimates for Germany in 2010 reported a share of 41% of indirect costs in total costs [12]. This gap is mainly due to the additional consideration of the economic consequences of unemployment in our work, which is generally not assessed in cost-of-illness studies. Łaszewska and colleagues also estimated costs due to unemployment and reported a share of 48% of unemployment excess costs in total excess costs, which aligns with our findings [13]. Without the consideration of unemployment (sensitivity analysis 2), the share of indirect costs in total costs decreased to 45%, which is consistent with earlier studies that have assessed indirect costs without unemployment [12, 62]. A literature review showed that the share of indirect costs in the total costs of schizophrenia was 44% on average [62]. However, the amount of indirect costs depends strongly on the level of disease severity, and could be more than twice as high at a high severity level (GAF < 50) than for a low severity level (GAF ≥ 70) [63]. These findings support the high share of indirect costs in our analysis, since over 90% of the individuals with schizophrenia, schizotypal and delusional disorders (ICD-10 F2) were in severity level 4 (GAF ≤ 50).

### Strengths and limitations

In this study, data from individuals with mental disorders were drawn from a RCT in the city of Hamburg in Northern Germany. A particular strength of this study was that the diagnoses were obtained in clinically structured interviews by experienced clinicians. This ensured higher data quality than with self-completed questionnaires, medical records or claims data from health insurance funds. The self-reported utilization of health care services and productivity losses were also assessed interview-based for a period of 6 months. For mental disorders in particular, it was shown that more cost categories were captured with survey data than with other data sources [64]. However, survey data are prone to recall bias and reporting bias, especially in the case of severe mental disorders. In addition, only complete cases were included in the analysis. In relation to the sample size, the proportion of excluded observations was higher in the group with severe mental illness than in the mild to moderate disease levels.

Another advantage of this study is that the individuals were insured in different major statutory health insurance funds, as differences between insurers were documented with regard to sociodemographic characteristics and morbidity [65]. However, not all statutory health insurance funds and no private insurance funds participated in the study, thus this study might be affected by selection bias. A large share of the individuals with mental disorders were recruited from the University Medical Center Hamburg-Eppendorf, which also poses the risk of selection bias (many severe cases and an underrepresentation of persons with undiagnosed mental disorders). In addition, due to the study design, not all diagnoses groups in the spectrum of mental disorders could have been included. On the one hand, persons with a primary diagnosis in the spectrum of organic, including symptomatic, mental disorders (ICD-10 F0) or mental and behavioral disorders due to psychoactive substance use (ICD-10 F1) were excluded due to the treatment options offered in the RECOVER study. On the other hand, the sample composition was open with regard to the diagnoses included, resulting in a heterogeneous group picture with a high proportion of mood disorders (ICD-10 F3), especially major depression. As a result, the representativeness of the study is limited, which limits the generalizability of our findings.

Another strength of this study was that the use of a comparison group allowed the calculation of excess costs, and thus a precise estimation of disease-related costs. We have balanced the control group using modern approaches. Yet, the data for the control group stem from another study setting and earlier survey period. The different interview modes might also have caused differences in the datasets. For example, it was shown that telephone interviews have a higher risk of social desirability bias than face-to-face interviews [66]. Moreover, it was not possible to estimate medication excess costs, as these were not collected in the control group. Despite for that, we have considered many cost categories and also evaluated unemployment in monetary terms, which has been rarely conducted for the evaluation of indirect costs. The high excess costs of unemployment showed that taking these costs into account for mental disorders is quite reasonable. However, the causal relationship of mental disorders with unemployment needs further clarification, since unemployment can also lead to mental disorders. Especially in the case of a mild disease severity, the causal relationship could be reversed, so that unemployment could have promoted the disease symptomology like depressive symptoms [67].

## Conclusions

The excess costs of mental disorders in our study were high and increased by levels of disease severity. Based on the study setting, our findings showed that acute phases of illness substantially increased the costs associated with mental disorders compared to population-based estimates. Future research is needed to investigate, whether these findings are also reflected in other diagnosis groups that are underrepresented in this study, as well as in larger study samples in Germany. Cost-of-illness studies serving as the basis for policy interventions and decisions for mental disorders should take indirect costs and the levels of disease severity into account.

## Supplementary Information

Below is the link to the electronic supplementary material.

Supplementary file1 (DOCX 39 KB)
