# Supplementary material for: Excess costs of mental disorders by level of severity
Source: Soc Psychiatry Psychiatr Epidemiol. 2022 May 31;58(6):973–85. doi: 10.1007/s00127-022-02298-8 (PMC10241728; doi:10.1007/s00127-022-02298-8)
Supplement: Supplementary file 1 — Supplementary file1 (DOCX 39 KB) [file 127_2022_2298_MOESM1_ESM.docx]

## Table S1 Overview of included primary diagnoses and diagnosis-specific questionnaires

| **Primary diagnosis** | **Diagnosis-specific questionnaires** |
| --- | --- |
| Mood disorders- Major Depression (ICD-10 F32-F39) | Patient Health Questionnaire (PHQ-9; Kroenke et al., 2001; German version Löwe et al., 2004) |
| Bipolar affective disorder  (ICD-10 F31) | The Altman Self-Rating Mania Scale (ASRM; Altman et al., 1997; German version Bräunig et al., 1996)  PHQ-9 |
| Agoraphobia/ Panic disorder [episodic paroxysmal anxiety] (ICD-10 F40.0/ F41.0) | Panic and Agoraphobia Scale (PAS; Bandelow, 1999) |
| Social phobias (ICD-10 F40.1) | Social Phobia Inventory (SPIN; Connor et al., 2000; German version Sosic et al., 2008) |
| Generalized anxiety disorder/ Specific (isolated) phobias (ICD-10 F41.1/ F40.2) | GAD-7 (Spitzer et al., 2006; German version Löwe et al., 2008) |
| Obsessive-compulsive disorder (ICD-10 F42) | Yale-Brown Obsessive Compulsive Scale (YBOCS; Goodman et al., 1989; German version Hand et al., 1991) |
| Post-traumatic stress disorder (ICD-10 F43.1) | PTSD Checklist for DSM-5 (PCL; Weathers et al., 2013; German version Krüger-Gottschalk et al., 2017) |
| Somatoform disorders (ICD-10 F45) | Patient Health Questionnaire 15 (PHQ15; Kroenke et al., 2002; German version Löwe et al., 2004) |
| Specific personality disorders  (ICD-10 F6) | PID-5 (German version Zimmermann et al., 2014) |
| Schizophrenia, schizotypal and delusional disorders  (ICD-10 F2) | Positive and Negative Syndrome Scale (PANSS; Kay et al., 1987) |
| Eating disorders  (ICD-10 F50) | Eating Disorder Examination–Questionnaire (EDE– Q Fairburn et al., 1994 ; German version Hilbert & Tuschen-Caffier, 2016) |
| Hyperkinetic disorders  (ICD-10 F90) | Adult ADHD Self-Report Scale (ASRS; Kessler et al., 2005) |

## Table S2 Unit costs

| **Cost category** | **Unit** | **Price per unit (2019)** |
| --- | --- | --- |
| **Outpatient healthcare services** |  |  |
| ***Physicians*** |  |  |
| Dermatologist | Per contact | 20.89 € |
| Dentist | Per contact | 61.78 € |
| General practitioner | Per contact | 22.18 € |
| Gynaecologist | Per contact | 33.32 € |
| Internist | Per contact | 72.36 € |
| Ophthalmologist | Per contact | 38.46 € |
| Orthopaedist | Per contact | 28.11 € |
| Otolaryngologist | Per contact | 29.19 € |
| Outpatient clinic/ Psychiatric outpatient department | Per contact | 41.13 €^1^ |
| Psychiatrist/ Neurologist | Per contact | 49.45 € |
| Psychologist/ Psychotherapist | Per contact | 86.33 € |
| Radiologist | Per contact | 41.13 €^1^ |
| Surgeon | Per contact | 47.98 € |
| Urologist | Per contact | 27.31 € |
| ***Other health-care professionals*** |  |  |
| Alternative practitioner/ Osteopath | Per contact | 33.18 € |
| Occupational therapist | Per contact | 41.45 € |
| Physiotherapy | Per contact | 18.16 € |
| Speech-language pathologist | Per contact | 42.67 € |
| **Inpatient healthcare services** |  |  |
| Day-care hospital | Per day | 244.13 € |
| General hospital | Per day | 636.72 € |
| Psychiatric hospital | Per day | 375.59 € |
| Rehabilitation | Per day | 134.72 € |
| **Social/ informal care services** |  |  |
| Home help | Per hour | 14.94 € |
| Informal care | Per hour | 19.87 € |
| **Productivity losses** |  |  |
| Average wage rate^2^ | Per day | 265.53 € |
| ^1^ Arithmetic mean of the unit costs of the other physicians, without dentists.  ^2^ Statistisches Bundesamt 2016, 2019.The published average wage rates were complemented with the employers social insurance contributions.  The unit costs reported were from Bock et al. (2015), Grupp et al. (2017) if not otherwise declared. | | |

## Table S3 Sample characteristics of the unmatched groups

| **Covariates** | **Individuals with mental disorders (n=816)** | **Unmatched control group without mental disorders (n=3,226)** |
| --- | --- | --- |
| **Age** (in years, mean) | 36.9 | 54.4 |
| **Gender** (female, %) | 59.9 | 53.1 |
| **Marital status** (%) |  |  |
| Single | 53.8 | 27.7 |
| Married/having a partner | 38.6 | 42.3 |
| Divorced/living separated | 7.6 | 30.0 |
| **School level** (%) |  |  |
| No school-leaving diploma | 3.4 | 1.0 |
| Special-needs school (Förderschule) | 0.5 | 0.0 |
| Secondary general school (Hauptschulabschluss) | 14.5 | 29.7 |
| Secondary school (Mittlerer Schulabschluss) | 28.9 | 35.1 |
| Academic secondary school ((Fach-)Abitur) | 52.7 | 34.2 |
| **Education level** (%) |  |  |
| No graduation | 37.0 | 10.8 |
| Completed vocational training | 40.7 | 65.2 |
| University diploma | 22.3 | 24.1 |
| **Body mass index** (%) |  |  |
| Underweight (<18.5) | 6.3 | 1.8 |
| Normal weight (18.5-24.9) | 52.8 | 46.1 |
| Overweight (25.0- 29.9) | 23.9 | 36.6 |
| Class I obesity (30- 34.9) | 11.2 | 11.6 |
| Class II-III obesity (≥35) | 5.9 | 3.9 |
| **Neoplasms**^1^ (%) | 1.6 | 9.0 |
| **Endocrine, nutritional and metabolic diseases**^2^ (%) | 15.2 | 30.7 |
| **Diseases of the circulatory system**^3^ (%) | 11.8 | 32.5 |
| **Diseases of the respiratory system**^4^ (%) | 16.3 | 14.8 |
| **Diseases of the digestive system**^5^ (%) | 6.9 | 16.4 |
| **Diseases of the skin and subcutaneous tissue**^6^ (%) | 5.9 | 13.1 |
| **Diseases of the musculoskeletal system & connective tissue**^7^ (%) | 13.2 | 31.1 |

^1^ ICD-10 chapter II

^2^ ICD-10 chapter IV

^3^ ICD-10 chapter IX

^4^ ICD-10 chapter X

^5^ ICD-10 chapter XI

^6^ ICD-10 chapter XII

^7^ICD-10 chapter XIII

## Table S4 Quantities of health care services used/productivity losses (mean, weighted)

| **Service utilization/productivity losses** | **Unit** | **All individuals with mental disorders (n=816)** | **Severity level 1 (n=56)** | **Severity level 2**  **(n=306)** | **Severity level 3**  **(n=294)** | **Severity level 4**  **(n=160)** | **Matched control group**^1^  **(n=816)** |
| --- | --- | --- | --- | --- | --- | --- | --- |
| Outpatient physicians | Contacts | 12.4 | 7.2 | 11.4 | 13.4 | 14.2 | 5.4 |
| Outpatient other healthcare providers | Contacts | 2.1 | 0.9 | 2.2 | 2 | 2.3 | 2.2 |
| Hospital | Days | 15.8 | 1.3 | 6.9 | 21.1 | 28.3 | 0.5 |
| Rehabilitation | Days | 0.7 | 0.7 | 0.8 | 0.6 | 0.7 | 0.2 |
| Social/ informal care | Hours | 22.3 | 18.3 | 12.1 | 29.1 | 30.8 | 8.3 |
| Sick leave | Days | 12.0 | 9.3 | 14.7 | 11.4 | 8.8 | 1.8 |
| Unemployment | % share of individuals | 28.9 | 14.3 | 13.7 | 36.7 | 48.8 | 2.9 |
| Early retirement | % share of individuals | 7.6 | 0 | 2.3 | 10.2 | 15.6 | 1.3 |

^1^ n=3,226 individuals in the control group were down weighted to match the individuals with mental disorders.

## Table S5 Mean costs of individuals with mental disorders by severity level (six months, in Euro 2019)

|  | **Severity level 1 (n=56)** | | **Severity level 2 (n=306)** | | **Severity level 3 (n=294)** | | **Severity level 4 (n=160)** | |
| --- | --- | --- | --- | --- | --- | --- | --- | --- |
| **Cost category** | **Mean costs** | **Standard Error** | **Mean costs** | **Standard Error** | **Mean costs** | **Standard Error** | **Mean costs** | **Standard Error** |
| Total costs | 8,664 | 1,593 | 12,383 | 881 | 26,724 | 1,179 | 34,425 | 1,510 |
| Direct costs | 1,253 | 486 | 3,160 | 375 | 8,366 | 701 | 11,228 | 1,022 |
| Outpatient physicians | 296 | 47 | 513 | 37 | 687 | 46 | 720 | 72 |
| Outpatient other healthcare providers | 17 | 7 | 49 | 8 | 54 | 10 | 64 | 19 |
| Hospital | 486 | 211 | 2,258 | 349 | 6,992 | 682 | 9,751 | 1,013 |
| Rehabilitation | 94 | 93 | 107 | 59 | 81 | 33 | 87 | 58 |
| Social/ informal care | 361 | 345 | 234 | 54 | 552 | 117 | 606 | 217 |
| Indirect costs | 7,411 | 1,543 | 9,223 | 723 | 18,358 | 882 | 23,197 | 1,089 |
| Sick leave | 2,480 | 459 | 3,893 | 409 | 3,036 | 414 | 2,324 | 479 |
| Unemployment | 4,931 | 1,614 | 4,738 | 679 | 12,680 | 971 | 16,828 | 1,364 |
| Early retirement | 0^1^ | - | 592 | 221 | 2,642 | 457 | 4,045 | 743 |

^1^ Only descriptive estimates are reported, because none of the individuals with mental disorders received a disability pension.

## Table S6 Sensitivity analysis 1: Unadjusted mean costs and excess costs of individuals with mental disorders (six months, in Euro 2019)

|  | **Individuals with mental disorders**  **(n=816)** | |  | **Unmatched control group**  **(n=3,226)** | | **Excess costs** | **95% confidence intervals** | **p-value** |
| --- | --- | --- | --- | --- | --- | --- | --- | --- |
| **Cost category** | **Mean costs** | **Standard error** |  | **Mean costs** | **Standard error** |  |  |  |
| Total costs | 21,617 | 703 |  | 3,021 | 145 | 18,596 | 17,190 – 20,002 | <0.001 |
| Direct costs | 6,487 | 373 |  | 1,231 | 73 | 5,256 | 4,511 – 6,001 | <0.001 |
| Outpatient physicians | 602 | 26 |  | 244 | 5 | 358 | 306 – 410 | <0.001 |
| Outpatient other healthcare providers | 51 | 6 |  | 69 | 4 | -17 | -31 – -3 | 0.016 |
| Hospital/day care | 5,311 | 360 |  | 523 | 49 | 4,788 | 4,077 – 5,500 | <0.001 |
| Rehabilitation | 92 | 28 |  | 65 | 11 | 27 | -33 – 87 | 0.374 |
| Social/ informal care | 430 | 68 |  | 330 | 38 | 100 | -52 – 252 | 0.198 |
| Indirect costs | 15,130 | 522 |  | 1,790 | 116 | 13,340 | 12,291 – 14,388 | <0.001 |
| Sick leave | 3,179 | 237 |  | 600 | 51 | 2,580 | 2,105 – 3,054 | <0.001 |
| Unemployment | 9,983 | 548 |  | 813 | 92 | 9,170 | 8,081 – 10,259 | <0.001 |
| Early retirement | 1,967 | 240 |  | 377 | 55 | 1,590 | 1,107 – 2,073 | <0.001 |

## Table S7 Sensitivity analysis 2: Mean costs and excess costs of individuals with mental disorders, without the costs of unemployment (six months, in Euro 2019)

|  | **Individuals with mental disorders**  **(n=816)** | |  | **Matched control group**  **(n=816^1^)** | | **Excess costs** | **95% confidence intervals** | **p-value** |
| --- | --- | --- | --- | --- | --- | --- | --- | --- |
| **Cost category** | **Mean costs** | **Standard error** |  | **Mean costs** | **Standard error** |  |  |  |
| Total costs | 11,633 | 529 |  | 1,554 | 184 | 10,079 | 8,982 – 11,176 | <0.001 |
| Direct costs | 6,487 | 373 |  | 743 | 157 | 5,744 | 4,950 – 6,537 | <0.001 |
| Outpatient physicians | 602 | 26 |  | 211 | 9 | 390 | 336 – 445 | <0.001 |
| Outpatient other healthcare providers | 51 | 6 |  | 49 | 5 | 2 | -13 – 18 | 0.768 |
| Hospital/day care | 5,311 | 360 |  | 292 | 122 | 5,020 | 4,275 – 5,764 | <0.001 |
| Rehabilitation | 92 | 28 |  | 28 | 13 | 64 | 3 – 125 | 0.040 |
| Social/ informal care | 430 | 68 |  | 163 | 29 | 267 | 123 – 412 | <0.001 |
| Indirect costs | 5,146 | 314 |  | 811 | 91 | 4,335 | 3,695 – 4,975 | <0.001 |
| Sick leave | 3,179 | 237 |  | 466 | 45 | 2,713 | 2,241 – 3,185 | <0.001 |
| Early retirement | 1,967 | 240 |  | 345 | 79 | 1,622 | 1,126 – 2,118 | <0.001 |

^1^ n=3,226 individuals in the control group were down weighted to match the individuals with mental disorders.
